# Is low dose inhaled corticosteroid therapy as effective for inflammation and remodeling in asthma? A randomized, parallel group study

**DOI:** 10.1186/1465-9921-13-11

**Published:** 2012-02-02

**Authors:** Melissa Baraket, Brian GG Oliver, Janette K Burgess, Sam Lim, Gregory G King, Judith L Black

**Affiliations:** 1 Woolcock Institute of Medical Research, Sydney, NSW, Australia; 2 Sydney Medical School, University of Sydney, Sydney, NSW, Australia; 3 Department of Respiratory Medicine, Liverpool Hospital, Sydney, NSW, Australia; 4 Department of Respiratory Medicine, Royal Prince Alfred Hospital, Sydney, NSW, Australia; 5 ANZAC Research Institute, Sydney, NSW, Australia; 6 The Cooperative Research Centre for Asthma, Glebe, NSW, Australia; 7 Department of Respiratory Medicine, Royal North Shore Hospital, Sydney, NSW, Australia

**Keywords:** asthma, corticosteroids, airway hyperresponsiveness, alveolar macrophage, airway inflammation, airway remodeling

## Abstract

**Background:**

While most of the clinical benefits of inhaled corticosteroid (ICS) therapy may occur at low doses, results of dose-ranging studies are inconsistent. Although symptom/lung function response to low and high dose ICS medication is comparable, it is uncertain whether low dose ICSs are as effective as high dose in the treatment of inflammation and remodeling.

**Methods:**

22 mild or moderate asthmatic adult subjects (corticosteroid free for > 2 months) participated in a randomized, parallel group study to compare effects of fluticasone propionate (FP) 200 mcg/day and 1000 mcg/day. Alveolar macrophage (AM)-derived cytokines and basement membrane thickness (BMT) were measured at baseline and after 7 weeks treatment while symptoms, spirometry, exhaled nitric oxide (eNO) and airway hyperresponsiveness (AHR) to mannitol at baseline and 6 weeks.

**Results:**

FP improved spirometry, eNO, symptoms and AHR with no difference between low and high dose FP. Both high and low dose FP reduced GM-CSF, TNF-alpha and IL-1ra, with no change in BMT and with no differences between low and high dose FP.

**Conclusions:**

200 μg/day of FP was as effective as 1000 μg/day in improving asthma control, airway inflammation, lung function and AHR in adults in the short term. Future studies should examine potential differential effects between low and high dose combination therapy (ICS/long acting beta agonist) on inflammation and airway remodeling over longer treatment periods.

## Background

Corticosteroids are the most effective medication for the prevention and treatment of asthmatic inflammation. Many dose-ranging studies have been conducted to examine the dose-response relationship of ICSs in the treatment of asthma in adults and children [1-17]. The adverse effects of inhaled corticosteroids (ICSs) have been clearly shown to be dose-related in both adults and children in numerous studies [18-25]. Although it has been suggested that most of the clinical benefits of ICSs occur at low doses [6,26], the results of dose-ranging studies are inconsistent. Some data show no dose-response for FEV1 or asthma symptoms [8-11,13,27-29], for airway hyperresponsiveness [7,14,15] or exhaled nitric oxide [15], while other data show a significant dose-response for clinical outcomes [2-5,7,9,12,16,17,30,31].

Evidence for equivalent effects on underlying airway inflammation and remodeling of low dose and high dose ICS therapy is lacking. *In vitro*, the degree of suppression of some inflammatory cytokines and the degree of upregulation of anti-inflammatory cytokines is dependent on the dose of corticosteroid [32,33]. However, there are no *in vivo *randomized studies comparing the effects of low and high dose ICS on production of clinically relevant cytokines.

Alveolar macrophages (AMs) normally represent the majority of cells isolated by bronchoalveolar lavage (BAL) and express allergen-specific low affinity immunoglobulin E (IgE) receptors on their surface [34] which, when activated, release pro-inflammatory mediators and anti-inflammatory regulators including chemokines (IL-8, MCP-1, MIP-1α), anti-inflammatory cytokines (IL-1ra and IL-10) and pro-inflammatory cytokines (TNF-α, GM-CSF, IL-6 and IL-1β) [35]. Endobronchial allergen challenge significantly increases expression of activation markers on AMs and correlates with AHR to methacholine [36]. The pro-inflammatory cytokine granulocyte macrophage-colony stimulating factor (GM-CSF), secreted by AMs, promotes myeloid cell differentiation, stimulates antigen presenting cells, prolongs eosinophil survival [37] and is elevated in BAL and bronchial biopsies in asthma and after allergen challenge [38-41]. Neutralisation by GM-CSF antibodies in mice abolishes ovalbumin-induced AHR and mucus cell hyperplasia [42]. *In vitro *incubation of peripheral blood mononuclear cell cultures with beclomethasone dipropionate (BDP) or fluticasone propionate (FP) dose-dependently inhibits allergen-induced GM-CSF in atopic asthmatics [32]. Tumour necrosis factor-alpha (TNF-α) is another pro-inflammatory mediator secreted by AMs which is increased in symptomatic asthmatics and enhanced by allergen challenge. *In vitro *incubation with TNF-α increases human airway smooth muscle responsiveness [43]. Inhaled recombinant TNF-α increases AHR to methacholine [44]. *In vitro *incubation of alveolar macrophages with fluticasone or budesonide results in dose-dependent inhibition of lipopolysaccharide (LPS)-induced TNF-α protein secretion [33].

AMs secrete the anti-inflammatory cytokine interleukin-10 (IL-10) which inhibits production of many pro-inflammatory cytokines [45], suppresses allergic inflammation by inhibiting T helper 2 cell cytokines [46], shortens eosinophil survival and attenuates induction of IgE synthesis by IL-4 [47]. Studies of the effects of corticosteroid treatment on IL-10 are mixed. IL-10 expression is greater during budesonide treatment compared with placebo [48] and serum IL-10 increases after triamcinolone [49] while inhibition of IL-10 by corticosteroid treatment can also occur [50].

Another anti-inflammatory cytokine secreted by AMs is interleukin-1 receptor antagonist (IL-1ra) which is an endogenous means of protection against inflammatory responses in diseases such as asthma and rheumatoid arthritis by inhibiting the pro-inflammatory mediator interleukin-1beta (IL-1β). More macrophages express IL-1β in asthma [51] and IL-1ra serum levels increase during asthma exacerbations [52]. In animal studies, aerosol IL-1ra prior to histamine challenge prevents bronchial hyperresponsiveness [53]. Inhaled BDP in asthmatic subjects inhibits epithelial expression of IL-1β without inhibition of IL-1ra, favouring an anti-inflammatory shift in the ratio of IL-1β/IL-1ra [54].

There is also some evidence for dose related differences in the effects of corticosteroids on bronchial mucosal basement membrane thickness (BMT) [55,56] but whether low dose ICS therapy, while sufficient to control asthma symptoms, is also adequate to improve airway remodeling is unknown.

The null hypothesis of this study is that low dose ICS therapy is as efficacious as high dose therapy, for asthma control, airway inflammation and remodeling in adults. Our aim was to examine differences in response to high and low dose ICS, in asthma control, airway inflammation, AHR and airway remodeling in mild to moderate adult asthmatics who were previously corticosteroid free for over two months.

## Methods

### Subjects

Adult volunteers were recruited, aged 19-30 years, with intermittent or mild persistent or moderate persistent atopic asthma (according to Global Initiative for Asthma, Global Strategy for Asthma Management and Prevention, NIH Publication, Updated 2005). Inclusion criteria were asthma symptoms in the preceding 12 months and positive mannitol bronchial provocation test. Exclusion criteria were baseline FEV1 less than 60% predicted, inhaled or systemic corticosteroid treatment in the preceding 2 months, smoking in the preceding 12 months, greater than 5 pack year smoking history and symptoms of upper respiratory tract infection in the past 6 weeks. The study was approved by the Ethics Review Committee of Royal Prince Alfred Hospital in the Sydney South West Area Health Service (protocol number X02-0137).

### Study design

This was a double-blind, randomized, parallel group comparison of short term low dose (100 μg twice daily) versus high dose (500 μg twice daily) inhaled fluticasone propionate (FP) via metered dose aerosol and large volume spacer (GlaxoSmithKline). Bronchoscopy with lavage and biopsies and bronchodilator reversibility assessment were performed before and after 7 weeks treatment. The other clinical measurements were obtained before treatment and one week prior to the second bronchoscopy, that is, after 6 weeks treatment. The canisters were weighed before and after the treatment period to confirm adherence. Patients were provided with salbutamol metered dose aerosol, 100 μg, used as needed for asthma symptoms. Skin prick testing was done with a panel of 12 common aeroallergens to assess atopic status. A positive wheal was 3 mm or more in the presence of a negative control.

### Lung function and airway hyperresponsiveness (AHR)

AHR was measured by bronchial provocation with dry powder mannitol [57]. The test was terminated when the fall in FEV1 reached at least 15% of baseline or after a cumulative mannitol dose of 635 mg [58]. Response dose ratio (RDR) [57] was the final percent fall in FEV1 divided by the corresponding cumulative dose of mannitol (%/mg). The provocative dose of mannitol causing a 15% or greater fall in FEV1 (PD15) was calculated by linear interpolation. The mannitol doubling dose difference (DDD(RDR)) was calculated as [log(RDR) pre FP - log(RDR) post FP]/log(2).

### Exhaled nitric oxide (eNO)

Exhaled air was collected, prior to any lung function tests, in a nitric oxide impermeable polyethylene reservoir bag using a slow vital capacity manoeuvre via a rotameter to ensure an expiratory flow rate of 200 mL/s. The fraction of eNO (ppb) was measured offline using a chemiluminescence analyzer (42C, Thermo Environmental Instruments, MA, USA).

### Bronchoscopy, alveolar macrophage (AM) culture and AM stimulation, RNA extraction and bronchial biopsy

Three endobronchial biopsies before and after 7 weeks of treatment were snap frozen in optimal cutting temperature medium and stored at -80°C for later sectioning. AMs were isolated from bronchoalveolar lavage fluid by adhesion. After rinsing and reapplication of culture medium, AMs were incubated with or without lipopolysaccharide (LPS) for 24 hours, after which supernatants were stored at -80°C for cytokine protein assay and AMs were harvested for RNA extraction.

### AM cytokine quantification

After reverse transcription, quantitative PCR for each cytokine was performed (Assays-on-Demand, Applied Biosystems, Foster City, California) to obtain a threshold cycle (Ct) with normalisation to 18S. Cytokine protein concentration in AM culture supernatants was quantified by enzyme-linked immunosorbent assay (ELISA) (DuoSet, R&D Systems, Minneapolis, USA).

### Basement membrane thickness (BMT)

Frozen biopsies were sectioned at -18°C at a thickness of 7 μm. Cryosections were stored at -80°C for later staining with haematoxylin and eosin which were then photographed under light microscopy at 60× power. Transverse measurements of the basement membrane, perpendicular to the mucosal surface, were taken at 10-20 μm intervals where the basement membrane appeared intact, clearly delineated and uniform. The mean BMT was calculated from seven to ten measurements on each of two to four sections cut from each of two to three biopsies.

### Statistical analysis

Statistical analyses were conducted using SPSS. Nonparametric variables (eNO, serum IgE, PD15, and RDR) were log transformed to normal distributions. Comparisons of means for normally distributed data (FEV1, BMT) were calculated by paired samples t-test for paired data and independent samples t-test for unpaired data. For Juniper Asthma Control Questionnaire scores [59], change in threshold cycle (ΔCt) and ELISA values which were non-normally distributed, medians were compared using the Wilcoxon test for paired samples and the Mann-Whitney U test for unrelated samples. Given the lack of available data quantifying the dose-dependent effects of inhaled corticosteroids on airway cytokines, the sample size was extrapolated from a study of the effects of inhaled budesonide on alveolar macrophage GM-CSF, TNF-alpha and IL-10 protein levels (pg/mL) [48] and a study of inhaled flunisolide on TNF-α protein [60]. A sample size of 11 subjects in each group was calculated to achieve a power of 80% and alpha of 0.05 to detect a difference of 1500 pg/mL in GM-CSF and 260 pg/mL in IL-10 based on a standard deviation of 1250 pg/mL for GM-CSF and 220 pg/mL for IL-10.

## Results

Twenty three subjects completed the protocol in full. Paired clinical and *in vitro *data were available for 22 participants, of whom 11 had been randomized to low dose and 11 to high dose FP.

### Baseline (pre FP) characteristics

Clinical baseline characteristics of the two treatment groups were not statistically different (table 1). Fifteen had reported symptoms and had used a rescue short-acting b2-agonist in the preceding 4 weeks, 21 in the preceding 6 months and all in the preceding 2 years. Sixteen subjects had taken ICSs previously (7 in low dose and 9 in high dose FP group) at or longer than 2.5 months prior to study entry, with a median interval since last corticosteroid use of 9 months. Twenty-two had never smoked, while 1 had a 0.2 pack year history and ceased 2 years prior. All subjects were atopic on allergen skin testing and had significant AHR to mannitol.

**Table 1:** Clinical baseline characteristics of the asthmatic subjects grouped into low and high dose FP treatment arms

	Low Dose FP (n = 11)	High Dose FP (n = 11)	
	**Mean (SD)**	**Mean (SD)**	**p value**

Age (yrs)	21.7 (2.9)	22.3 (4.0)	0.72
Height (m)	1.7 (0.1)	1.8 (0.1)	0.24
Weight (kg)	74.1 (15.7)	86.8 (24.0)	0.16
Age of asthma onset (yrs)	6.7 (2.8)	4.8 (4.2)	0.22
Duration of asthma (yrs)	14.5 (4.0)	16.5 (6.1)	0.37
FEV1 (L)	3.49 (0.56)	3.63 (0.74)	0.63
FEV1 % predicted	85.6 (12.6)	84.5 (12.1)	0.84
FVC (L)	4.59 (0.87)	4.79 (0.86)	0.60
FEV1/FVC ratio (%)	77.4 (12.3)	75.9 (7.3)	0.74
Bronchodilator response (%)	5.6 (6.5)	7.0 (4.0)	0.54

	**Geometric Mean** **(95% GR)**	**Geometric Mean** **(95% GR)**	**p value**

Exhaled nitric oxide (ppb)	22.5 (5.7-88.5))	24.6 (5.7-106.6)	0.77
Total serum IgE (kU/L)	246.7 (19.4-3142.4)	411.7 (34.7-4884.3)	0.36
PD15 (mg)	178.0 (29.3-1080.2)	96.8 (20.1-465.8)	0.11
RDR (%/mg)	0.087 (0.015-0.478)	0.145 (0.030-0.668)	0.16

	**Median (IQR)**	**Median (IQR)**	**p value**

Juniper ACQS	1.0 (0-2.0)	*1.0 (0-2.0)	0.67

GR = geometric range, IQR = interquartile range, *n = 10

### Comparison of the change in each endpoint between low and high dose FP

There were no significant differences in mean or median changes in any clinical parameters between low and high dose FP groups (table 2). No statistically significant differences were found in median changes in AM cytokine mRNA expression or protein production between low and high dose FP groups. The difference in mean changes in BMT between low and high dose FP was also not statistically significant.

**Table 2:** Change in each endpoint after FP in low and high dose treatment arms

	Low dose FP (n = 11)	High dose FP (n = 11)	
	**Mean (SD)**	**Mean (SD)**	**p value**

change in FEV1 (L)	0.291 (0.512)	0.364 (0.356)	0.703
change in percent predicted FEV1 (%)	6.5 (11.1)	8.0 (7.1)	0.719
change in FEV1/FVC ratio after FP (%)	3.5 (4.6)	5.7 (6.1)	0.336
change in Bronchodilator response (%)	0.1 (7.8)	2.7 (5.5)	0.376
change in log RDR (%/mg)	0.955 (0.530)	0.751 (0.433)	0.335
change in log exhaled NO (ppb)	0.307 (0.259)	0.427 (0.304)	0.331
Mannitol Doubling Dose Difference	3.2 (1.8)	2.5 (1.4)	0.335
change in basement membrane thickness (μm)	-0.27 (1.08)	0.56 (1.38)	0.133

	**Median (IQR)**	**Median (IQR)**	**p value**

change in Juniper ACQS	0.5 (0-1.0)	1.0 (0-1.0)	0.969
change in LPS induced TNF-alpha mRNA (Ct)	0.1 (-0.7-1.7)	0.7 (-0.4-2.1)	0.797
change in LPS induced GM-CSF mRNA (Ct)	1.7 (-1.4-3.8)	2.9 (-1.9-5.0)	0.748
change in LPS induced IL-1ra mRNA (Ct)	3.2 (2.0-4.3)	3.3 (1.3-5.7)	0.606
change in LPS induced IL-10 mRNA (Ct)	-0.6 (-1.2-1.7)	0.5 (-0.7-1.2)	0.332
change in LPS induced TNF-alpha protein (pg/mL)	830 (-3888-1302)	-2860 (-9173-2716)	0.395
change in LPS induced GM-CSF protein (pg/mL)	307 (-414-2986)	418 (-619-1154)	0.768
change in LPS induced IL-1ra protein (pg/mL)	4035 (1493-58151)	10166 (-1631-15164)	0.922
change in LPS induced IL-10 protein (pg/mL)	0.5 (-75.6-19.2)	1.3 (-44.5-79.7)	0.622

SD = standard deviation, IQR = interquartile range, Ct = threshold cycle

### Effects of FP on clinical, physiological and in vitro parameters

**Figure 1:**
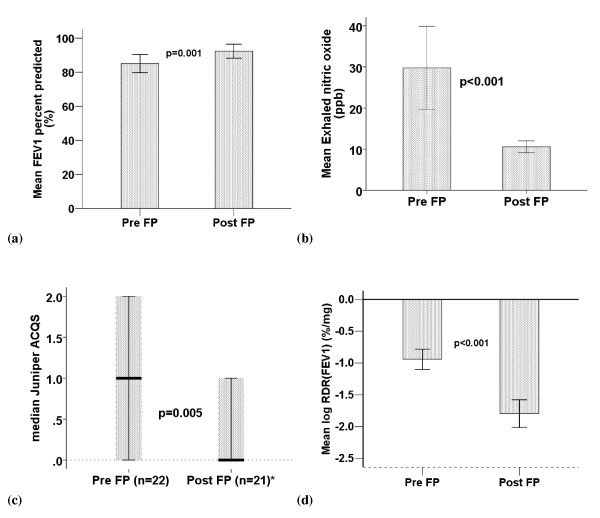
**Effects of FP on clinical parameters (n = 22)**. (a) FEV1 % predicted, (b) Exhaled nitric oxide (p value derived from log eNO means), (c) Juniper ACQS, (d) log RDR for mannitol. Columns represent means and error bars represent 95% confidence intervals. Juniper scores are depicted by box and whisker plots with medians and interquartile ranges (*n = 21 for post Juniper ACQS).

Because no significant differences were found between the low and high dose treatment groups, the effect of FP on the 22 asthmatics as a single group was evaluated. As shown in Figure 1, FP resulted in significant improvement in FEV1 % predicted, eNO and Juniper symptom score in this group of asthmatic subjects. There was also marked attenuation of AHR manifest by a significant fall in RDR after FP. Significant improvements were also demonstrated in FEV1 [pre FP: 3.56L (SD 0.64), post FP: 3.89L (SD 0.70), p = 0.002] and FEV1/FVC ratio [pre FP: 77% (SD 9.9%), post FP: 81% (SD 7.4%), p = 0.001].

**Figure 2:**
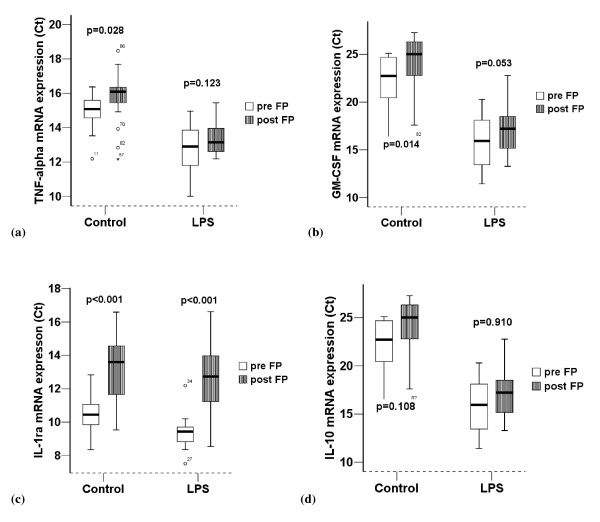
**Comparison of cytokine mRNA expression pre and post FP (n = 22)**. (a) TNF-α, (b) GM-CSF, (c) IL-1ra, (d) IL-10. Results are normalized for 18S. Ct = threshold cycle. LPS = Lipopolysaccharide. Control = unstimulated sample. Graphical representation is by box and whisker with medians and interquartile ranges. Note: an increase in threshold cycle indicates a decrease in quantity of mRNA present.

**Figure 3:**
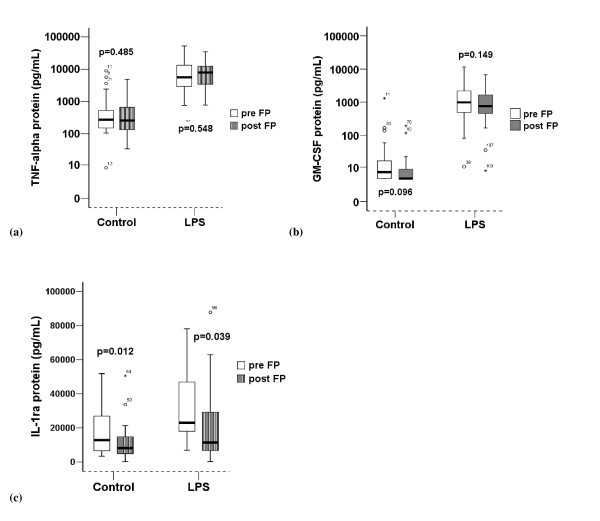
**Comparison of cytokine protein concentrations pre and post FP (n = 22)**. (a) TNF-α, (b) GM-CSF, (c) IL-1ra. LPS = Lipopolysaccharide. Control = unstimulated sample. Graphical representation is by box and whisker with medians and interquartile ranges.

LPS significantly enhanced the quantity of cytokine mRNA expressed and protein produced by AMs. FP treatment significantly reduced constitutive mRNA expression of GM-CSF, TNF-α and constitutive or LPS induced IL-1ra mRNA (Figure 2) and protein (Figure 3). There was no significant effect of inhaled FP on GM-CSF or TNF-α protein concentrations. There was no significant effect of inhaled FP on IL-10 which was in many samples below the lower limit of detection.

There was no significant change in BMT in the group as a whole (n = 22), 9.21 μm (SD 1.02) before and 9.06 μm (SD 0.91) after FP treatment.

## Discussion

In this study we found that seven weeks of low dose ICS treatment in adults with mild to moderate asthma, who were corticosteroid free for over 2 months, is as effective in controlling airway inflammation as high dose therapy, measured by constitutive mRNA expression of GM-CSF, TNF-α and constitutive or LPS induced IL-1ra mRNA and protein in BAL. Low and high doses were also equally effective in controlling asthma in terms of symptoms, spirometry, eNO and AHR after 6 weeks, which is consistent with previous data [6,26]. We found no significant effects on airway remodeling by either dose after 7 weeks, as measured by change in basement membrane thickness.

There have been previous ICS dose-ranging trials which have not found differential clinical effects between dose arms. Meta-analyses have revealed plateaus in the ICS dose-response curve beginning at 100-200 μg of FP per day with a peak benefit at 500 μg/day [11] or most of the clinical efficacy attained with an FP dose of 200 μg/day [6] or 300-600 μg/day of budesonide (BUD) [26]. However, control of underlying inflammation and remodeling was not assessed and individual patients with more severe asthma may require higher doses to achieve the corresponding maximal effects. Previously published evidence suggests that AHR, which is loosely related to inflammation, may be more likely to show ICS dose-dependence than FEV1, PEF or symptoms [9,12,31,61] and a dose-dependent rate of reduction of eNO and asthma symptoms has been observed [7,16,17]. In a meta-analysis of sixteen trials using multiple drug formulations (FP, triamcinolone, BUD and mometasone), a statistically significant inhaled corticosteroid dose-response relationship was found for morning PEF and a dose-response relation for FEV1 was found for BUD [2]. Asthma symptom score was also differentially affected by the dose of FP or triamcinolone, in contrast to the present study finding of no difference in change in Juniper score between the two doses. This absence of difference in the present study may be due to a milder degree of asthma and smaller sample size.

Despite the small sample size in a study by Chetta et al. [56] (8 in each group), there were significant differences in a number of *in vitro *inflammatory indices between low and high dose inhaled steroid treatment. High dose treatment resulted in a significant decrease in basement membrane thickness, number of vessels, vascular area and mean vessel size whereas no change was observed with low dose. Like the current study findings, there were no significant differences in the improvements in AHR and asthma symptom scores between the groups. The lack of difference between high and low dose ICS effect on inflammatory markers in the present study, compared with the findings of Chetta et al. [56] could be due to milder asthma in our subjects since none had previously taken long term ICS regularly. This is supported by the correlation observed between baseline severity and magnitude of improvement in outcomes.

Modulation of alveolar macrophage inflammatory responses is an important mechanism underlying ICS efficacy in asthma [34-36,62-65]. There are no published randomized studies examining the dose-response relationship of the effect of ICSs on pro- and anti-inflammatory cytokine production in asthma. In the present study inhaled FP significantly reduced mRNA expression of the pro-inflammatory cytokines GM-CSF and TNF-α. The change in GM-CSF protein levels after FP treatment was smaller than the estimated effect size of 1500 pg/mL. This estimate was derived from a study which had assessed the effect of inhaled budesonide (800 μg twice daily) in comparison to placebo [48], given the lack of available data quantifying the dose-dependent effects of inhaled corticosteroids on airway cytokines. A smaller effect size would be expected between two active treatment arms of different doses than between active treatment and placebo but, while this difference may be considered clinically significant, a greater number of participants in each group would have been required to show statistical significance. The effect size from two previous studies which measured alveolar macrophage TNF-α protein reduction post inhaled flunisolide [60] (in the order of 50 pg/mL) differed greatly compared with post inhaled beclomethasone [66] (in the order of 140,000 pg/mL). Therefore, although TNF-α is an important biological endpoint in this study, the effect of inhaled steroid treatment on TNF-α may depend on factors that are, as yet, undefined making the lack of significant effect of FP on TNF- α protein in the present study difficult to interpret. Inhaled or systemic corticosteroids suppress inflammatory cytokines *in vitro *including GM-CSF or TNF-α [40,48,66-73]. Previous *in vitro *studies have shown that inhibition of pro-inflammatory cytokines by corticosteroids is dose-dependent [32,33] and it was postulated that the *in vivo *effects of treatment on the same inflammatory markers would also be dose-dependent. The present study, however, found no significant difference between low and high dose inhaled FP groups in the change in quantity of constitutive and LPS induced cytokine mRNA and protein from alveolar macrophages.

The anti-inflammatory cytokine IL-1ra mRNA expression and IL-1ra protein was also significantly suppressed by inhaled FP in the present study. Previous studies examining the effects of ICSs on IL-1ra show conflicting results ranging from no effect [54,74] to elevation of IL-1ra levels post treatment [52,74]. In the current study, the finding of a reduction in IL-1ra mRNA and protein after treatment with FP was unexpected considering this cytokine has anti-inflammatory properties. Epithelial expression of IL-1β is significantly inhibited by inhaled BDP in asthmatic subjects without any significant concurrent inhibition of IL-1ra thus favouring an anti-inflammatory shift in the ratio of IL-1β/IL-1ra [54]. It is possible that the pro-inflammatory cytokine IL-1β, which was not measured in this study, may have been attenuated by FP to a greater extent than was IL-1ra, thus resulting in a lower, more anti-inflammatory ratio of IL-1β to IL-1ra levels.

The present study found no significant changes in the anti-inflammatory cytokine IL-10 gene expression or protein secretion resulting from treatment with FP. The small differences in IL-10 protein levels detected after FP treatment were substantially smaller than the 260 pg/mL reported previously. The levels of IL-10 were close to the lower limit of detection in many subjects making resolution of changes impossible. Previous studies have revealed mixed effects of corticosteroids on IL-10, including no effect of *in vitro *dexamethasone [75], elevation after high dose inhaled budesonide [48] or inhaled triamcinolone [49] and inhibition in nonasthmatics [50].

Basement membrane thickness in asthma is reduced by corticosteroid treatment in some studies [73,76-80]. Although dose related differences in the effects of corticosteroids on bronchial mucosal BMT are apparent in some studies [55,56], the current study did not demonstrate a significant reduction in BMT after FP treatment for 7 weeks. This is consistent with the findings of Boulet et al. for high dose FP [81]. Although two previous studies have shown a significant effect of short term treatment (six weeks) with medium or high dose inhaled corticosteroid on BMT [56,76], longer treatment duration (three to twelve months) is more likely to alter BMT [73,77,78,82-84].

## Conclusions

An important feature of current asthma management guidelines is the use of a maintenance dose of ICS which is the minimum effective dose to achieve asthma symptom control to limit the potential for adverse effects. Current therapy guidelines also advocate alternative approaches to achieve asthma control without increasing the ICS dose. The addition of a long-acting beta agonist, leukotriene antagonist and/or low dose theophylline represent examples of this approach. At least on a short term basis, our results suggest that in stable mild or moderate adult asthmatic patients, who are corticosteroid free for over 2 months, an FP equivalent dose of 200 μg/day may be just as effective as 1000 μg/day in controlling cytokine driven inflammation. Combining the different anti-asthmatic mechanisms of other therapies with the attenuation of cytokine driven inflammation by ICS might be a superior and safer approach to achieving asthma control.

It is possible that differences in inflammation may occur with longer term treatment and it would be important to conduct longitudinal studies with larger sample size to re-evaluate the dose-response relationship of a wide repertoire of inflammatory mediators, along with the probably smaller influence of dose on remodeling and fibrotic markers. Additionally, prevention of asthma exacerbations and decline in lung function would be important endpoints to assess in a long term study. It would also be useful to assess any differential dose effects of combination inhaled corticosteroid/long acting beta-agonist maintenance treatment, to firmly establish whether such low dose therapy optimally suppresses underlying inflammation and prevents long term airway remodeling and not just short term symptoms.

## Competing interests

Between 2006-10, Dr Gregory King has received various travel sponsorships from Boehringer Ingelheim, Pfizer, AstraZeneca and GlaxoSmithKline for travel and accommodation to attend international, local and interstate meetings that include Pharmaceutical Industry sponsored meetings and independent Society Scientific meetings. A proportion of Dr King's research work is conducted at the Woolcock Institute of Medical Research, which receives unrestricted grants from Boehringer Ingelheim, AstraZeneca and GlaxoSmithKline, Pharmaxis and which also has current and past consultancy agreements with Pfizer, Boehringer Ingelheim, AstraZeneca and GlaxoSmithKline. Dr King provides consultancy services related to asthma and COPD, which include sitting on advisory boards and providing talks at local and national meetings. His research group receives a proportion of the grants and monies that arise from those companies, as part of a general allocation of those funds for research purposes across all research groups of the Woolcock Institute of Medical Research. Dr King's research group is supported from competitive grants arising from local research foundations, the National Health & Medical Research Council of Australia, Cooperative Research Centre for Asthma and Airways and The Australian Lung Foundation. The other authors declare that they have no competing interests.

## Authors' contributions

MB contributed to study design, conducted the study, clinical tests and laboratory experiments and drafted the manuscript. BGGO helped with some experiments and provided laboratory technical advice, JKB provided laboratory technical advice, SL conceived and designed the study, GGK contributed to study design and provided clinical physiology technical advice, JLB contributed to study design and provided scientific advice. All authors were involved in discussion and interpretation of results and all authors critically revised the intellectual content of the manuscript. All authors read and approved the final manuscript.
